# Teamwork in community health committees: a case study in two urban informal settlements

**DOI:** 10.1186/s12913-023-10370-5

**Published:** 2023-12-07

**Authors:** Robinson Karuga, Sitara Khan, Maryse Kok, Malkia Moraa, Patrick Mbindyo, Jacqueline Broerse, Marjolein Dieleman

**Affiliations:** 1 LVCT Health, P.O. Box 19835, Nairobi, 00202 Kenya; 2 Athena Institute, Vrije Universiteit Amsterdam, De Boelelaan 1085, Amsterdam, 1081 HV The Netherlands; 3 KIT Royal Tropical Institute, Mauritskade 64, 1092 AD Amsterdam, Netherlands; 4 Directorate of Preventive and Promotive Health, Nairobi City County, City Hall Way, P.O Box 30075-00100, Nairobi, Kenya; 5 Jomo Kenyatta University of Agriculture and Technology, P.O. Box 62 000, Nairobi, 00200 Kenya

**Keywords:** Community health committees, Community health services, Community participation, Community health promoters, Kenya, Teamwork

## Abstract

**Background:**

Community health committees (CHCs) are mechanisms for community participation in decision-making and overseeing health services in several low-and middle-income countries (LMICs). There is little research that examines teamwork and internal team relationships between members of these committees in LMICs. We aimed to assess teamwork and factors that affected teamwork of CHCs in an urban slum setting in Nairobi, Kenya.

**Methods:**

Using a qualitative case-study design, we explored teamwork of two CHCs based in two urban informal settlements in Nairobi. We used semi-structured interviews (*n* = 16) to explore the factors that influenced teamwork and triangulated responses using three group discussions (*n* = 14). We assessed the interpersonal and contextual factors that influenced teamwork using a framework for assessing teamwork of teams involved in delivering community health services.

**Results:**

Committee members perceived the relationships with each other as trusting and respectful. They had regular interaction with each other as friends, neighbors and lay health workers. CHC members looked to the Community Health Assistants (CHAs) as their supervisor and “boss”, despite CHAs being CHC members themselves. The lay-community members in both CHCs expressed different goals for the committee. Some viewed the committee as informal savings group and community-based organization, while others viewed the committee as a structure for supervising Community Health Promoters (CHPs). Some members doubled up as both CHPs and CHC members. Complaints of favoritism arose from CHC members who were not CHPs whenever CHC members who were CHPs received stipends after being assigned health promotion tasks in the community. Underlying factors such as influence by elites, power imbalances and capacity strengthening had an influence on teamwork in CHCs.

**Conclusion:**

In the absence of direction and support from the health system, CHCs morph into groups that prioritize the interests of the members. This redirects the teamwork that would have benefited community health services to other common interests of the team. Teamwork can be harnessed by strengthening the capacity of CHC members, CHAs, and health managers in team building and incorporating content on teamwork in the curriculum for training CHCs.

## Background

Participation of community members in the design and delivery of primary health services is a cornerstone of primary health systems [1–3]. Communities that receive primary health services are expected to actively participate in governance (making and shaping decisions that affect their health and lives) of these services [4]. Lay community members can participate in the governance of primary health services through mechanisms such as suggestion boxes and health committees (Brinkerhoff 2003). Health committees are mechanisms through which community members can participate in governing primary health services. A health committee is defined as *“any formally constituted structure with community representation that has an explicit link to a health facility and whose primary purpose is to enable community participation in health with the aims of improving health service provision and health outcomes”* [5] pg. 451).

Specific roles and responsibilities of health committees vary depending on country and/or community context, where they are expected to accomplish their objectives as a team ([6], pg. 82). A *team* can be defined as an identifiable social group that is made up of individuals who are dependent on each other and work towards a common goal, which could not be accomplished by a single person [7–9]. Individuals in a team need to cooperate in performing the mutually agreed tasks to achieve team goals [7]. *Teamwork* is a set of attitudes (belief in the collective ability of the team and in the need to work together); and behaviors (communicating, sharing information, performing tasks together and checking for mutual understanding) that teams use to coordinate and collaborate their efforts to achieve their goals [7, 9]. Teamwork in primary health care has shown to be beneficial in distributing workload among health workers, reinforcing individual capabilities, generating a diversity of ideas, and creating a feeling of contribution (participation) and involvement [10]. Teamwork among health professionals has proven to be essential for delivering high quality of care to patients [11], making better decisions, and making complex tasks easier, while being cost-effective [12–14].

Research on teamwork among health workers in low- and middle-income countries (LMICs) is limited. A few studies on teamwork have been conducted in clinical care settings [15]. There is also lack of a conceptual foundation for researching teamwork in community governance structures. The factors that influence teamwork in community governance structures, such as health committees, are not well understood.

Community members in Kenya are expected to participate in providing oversight over health services that are offered at the household level by Community Health Promoters (CHPs) [16, 17]. CHPs are nominated for this role by fellow residents during community dialogue meetings. CHPs are then trained on how to deliver basic health-promotion services and referrals at community level. CHPs serve on a voluntary basis, but several devolved sub-national governments (counties) have formulated policies to provide financial incentives to CHPs. CHPs are supervised by community health assistants (CHAs). CHAs are trained public or environmental health workers employed by county governments. Community health services are delivered in geographical units referred to as community units. Each community unit has an estimated population of 5,000 people and is expected to be served by ten CHPs, under the supervision of five CHAs [18]. Community health services in each community unit are to be overseen by community health committees (CHCs), which are supposed to have between 11 and 13 volunteers, chaired by a lay-community member. Each CHC is expected to have a maximum of two CHPs. CHAs are the designated secretaries of the CHC. CHCs are institutionalized in Kenya’s Community Health Strategy as a mechanism for providing community oversight over the delivery of community health services [18]. The functions of the CHCs are as follows (pg. 948) [19]:

(a) Providing leadership and oversight in the implementation of health and other related community services (b) Coordinating the selection of CHPs within the community health unit through public participation forums (c) Preparing and presenting the community health unit's annual and operational work plans to the link facility health committee. (d) Planning, coordinating, and conducting the quarterly community dialogue and monthly health action days (e) Collaborating with the link facility to promote facility accountability to the community (f) Holding quarterly consultative meetings with the link facility (g) Creating an enabling environment for implementing community health services, and (h) Mobilizing resources for sustainability.

The objective of this study was to assess teamwork within CHCs to provide insights into team attitudes and behavior, which could inform the formation, training and management of community-level health committees in Kenya.

## Methods

### Study design and setting

We used a qualitative case study design to allow for an in-depth exploration of the interactions between CHC members and identification of factors that influence collaboration between individual team members and the overall teamwork in CHCs. The informal settlements we selected for this research are located in the eastern part of Nairobi City County and have an estimated population of 575,871 [20]. The study was conducted in two urban informal settlements to provide a broad spectrum of factors influencing teamwork in CHCs. We conduct this study in urban informal settlements because teamwork in community level health teams had been explored previously in rural settings [10, 21]. The study sites are served by 370 CHPs supervised by only three [3] CHAs and 74 CHC members across 37 community units [22].

We adopted the theoretical framework developed by Yeboah-Antwi et al. (2013) to qualitatively assess teamwork among CHCs. This framework comprises eight processes and 17 factors that are essential for community-based health workers to effectively work together as teams [10] (Table 1).

**Table 1:** Processes and factors that influence teamwork [10]

Teamwork **Process**	**Factors of teamwork**
1. Mutual performance monitoring	a) Consulting each other b) Seeking help from each other c) Checking each other’s work and giving feedback
2. Mutual trust	d) Confidentiality e) Respect f) Trust
3. Decision making/planning	g) Making decisions together h) Making a plan together i) Dividing tasks so as not to duplicate effort
4. Team cohesion	j) Interest and commitment k) Members available and accessible
5. Team motivation	l) Motivating each other m) Encouraging each other
6. Goals and objectives	n) Having a common goal
7. Communication	o) Good communication p) Sharing information
8. Conflict resolution	q) Ability to manage conflict

### Sampling

The sub-county-level primary health services manager and CHAs in our study setting categorized CHCs as either “well-functioning”, “moderately functioning” or “dysfunctional”. “Well-functioning” CHCs met three criteria: (1) regularly mobilized community members for dialogue days, (2) actively supervised CHPs, and (3) ensured timely submission of monthly household reports by CHPs to the CHAs. “Moderately functioning” were those CHCs that only met one of the three criteria. We excluded dysfunctional CHCs because, in our judgement, they would be difficult to approach and include in this study within the limited time available. With guidance from health managers in our study setting, we sampled one “well-functioning” and one “moderately functioning” CHC. After sampling, the health managers and two authors (SK and RK) met with the CHC chairpersons to brief them on the study. CHC chairpersons then informed their members that we were recruiting participants to take part in semi-structured interviews (SSIs) and group discussions.

### Data collection

#### We collected data in two phases

The first phase involved SSIs and group discussions. SSIs were conducted with 16 CHC members: ten (6 female, 4 male) in the well-functioning CHC, and six (5 female, 1 male) in the moderately functioning CHC. During the SSIs, we applied graphic elicitation techniques to facilitate probing. We asked participants to visually represent their experiences, beliefs and perceptions about interactions between CHC members, decision-making processes and communication channels using relationship maps on a flip-chart paper [23]. We then used the relationship maps to probe about topics such as respect, trust, cohesion, motivation and team cohesion. We found this technique helpful in enabling participants to narrate their attitudes and behavior related to teamwork and interactions with fellow CHC members [23]. On average, SSIs took 45 min. We also conducted two group discussions with the same CHC teams. We explored topics on their understanding of teamwork to deliver according to their roles and responsibilities; how clarity about their own roles influenced teamwork, and conflict resolution during the group discussions. The group discussions with the CHC teams also triangulated the data collected during SSIs with individual CHC participants. To prevent response bias, which may have been caused by the presence of CHAs, during the group discussions, we requested the CHAs not to participate. The group discussion with the “well-functioning” CHC had six participants (2 male, 4 female) and the discussion with the “moderately functioning” CHC had four participants (one male, three female). Both group discussions lasted 30–40 min.

We conducted the SSIs and group discussions between April and May 2019 in private spaces within the community. We conducted interviews with the CHAs in English, while interviews with other CHC members and group discussions were conducted in local Swahili colloquial language. Throughout the study, two authors (SK and RK) took field notes to document observations during the SSIs and group discussions.

### Data analysis

The anonymized audio-recordings of SSIs and group discussions were transcribed verbatim by a third-party transcriber for the interviews conducted in Kiswahili and by the co-lead author (SK) for those conducted in English. Transcripts in Kiswahili were translated into English by experienced bi-lingual translators before analysis. The second co-author (RK) validated the translated transcripts by reading them while listening t the audio recording to ensure that no meaning was lost during the translation. The transcripts from Microsoft Word (™) were imported into Atlas.ti for coding, text search and retrieval. We analyzed transcripts using the thematic analysis method to identify, analyze, organize and describe the themes that emerged from our SSIs and group discussions [24, 25]. The lead authors (RK and SK) read through all the SSI and group discussion transcripts and deductively populated each theme with narratives from the SSIs and group discussions and then wrote analytical summaries that described the emerging evidence from data in each theme, as well as including emerging themes that appeared throughout the study.

The a priori themes were based on the eight processes of teamwork in the conceptual framework. While analyzing the data, we combined some components of the a priori framework to eliminate redundancy. Participants conflated discussions related to mutual performance monitoring and communication. We made a similar observation when participants discussed team cohesion. In order to enhance the richness of the analyses, we presented data on “mutual performance monitoring and communication” and “cohesion and collectiveness”. We therefore coded our data into seven themes. Three authors further reviewed the coded data, independently, as part of quality assurance (RK, MD and MK), who also participated in writing up the results. There was consensus about the data coding among all the co-authors who independently reviewed the coded data.

### Reliability, rigor and credibility

We made efforts to enhance the trustworthiness of our findings by triangulating them through a 60-min group discussion with four CHAs (two male and two female), who were not part of the study's initial phase. The aim of this discussion was not only to triangulate our findings but also to increase the credibility and transferability of our study. We shared our preliminary analysis with the CHAs and asked for their interpretations of the findings as well as recommendations based on them, as part of the validation process. We also triangulated data from the SSIs with CHC members by conducting two group discussions with the same CHC members. Throughout the data collection and analysis processes, four authors (SK, RK, MD, and MK) held regular debrief sessions. SK and RK also held reflexivity sessions at the end of each fieldwork day, after data collection, to reflect on how their personal beliefs and positions as researchers may have influenced their interactions with participants and interpretation of the data [26]. During the data analysis, all authors discussed the coded data and interpreted the key narratives.

### Ethical considerations

Ethics clearance to conduct this study was obtained from the Kenya Medical Research Institute Ethics Review Committee (Non-SSC Protocol No.144). We safeguarded the autonomy of our research participants by obtaining written informed consent prior to conducting SSIs and group discussions. Participants in group discussions were requested to keep information shared during discussions confidential and not share them with others beyond the study, as they signed the informed consent forms. We made sure that any identifiers used in the visualized exercise were anonymized to protect the identities of persons who may have been listed on the flip charts. We received administrative clearance to conduct this research from the Nairobi City County Research Committee.

## Results

We will start this section by presenting the characteristics of CHCs, followed by the analysis of teamwork based on the Yeboah et al.’s theoretical framework [10].

### CHC characteristics

The well-functioning CHC had a total of nine members who had a median age of 46 years (range: 30 to 66 years). The moderately functioning CHC had six members with a median age of 41 years (range: 29 to 57 years). None of the CHC members were clear on how long the CHCs had been in existence because they joined their teams at different times. Most CHC members in both CHCs had attained primary and secondary education, and were involved in micro-enterprises within the informal settlements. Many CHC members held other leadership roles within their communities, such as youth group leaders and church officials. All participants reported being trained on communication skills, how to supervise CHPs, and how to solve conflicts when they joined the CHC. They were, however, not clear in which year they received these trainings and they did not have any documentation to prove they participated in the training. Detailed CHC characteristics are provided in Table 2.

**Table 2:** Characteristics of CHCs in this study

	**Well-functioning CHC (*N* = 10)**	**Moderately well-functioning CHC (*N* = 6)**
Median age	46 years (Range: 30 to 66)	41 years (Range: 29 to 57)
Average number of years as members in the study CHCs	2.4 years	2.1 years
Gender of the CHC Chairperson	Female	Male
**Gender**		
Male	4	1
Female	6	5
**Education status**		
Primary	3	1
Secondary	4	2
Tertiary	3	3
**Occupation**^a^		
Micro-enterprise	4	3
CHP	5	4
CHA	1	1
CHC members who had other leadership roles in the community *(village elders, church, leaders youth leaders *etc*.)*	3	5

^a^ N.B.: some participants had more than one occupation

Chairpersons in both CHCs were elected by the community and not all roles in the teams were consistent with the ones stipulated in the community health strategy. For example, the moderately functioning CHC had a vice-chairperson and an assistant secretary. Both of these roles do not exist in the community health strategy. The chairperson of the well-functioning CHC also served as the CHC secretary, which is inconsistent with the Ministry of Health guidelines on CHC composition:

“My role [in the CHC] should be [that of] the Secretary. […] But in reality, you find I am acting as the Chair and the Secretary. […] I am forced to come up with the agenda [which] should come from the Chair. […] I also convene our meetings.” (Male community member, well-functioning CHC, SSI)

### Communication and mutual performance monitoring

In this theme, we explored how CHC members consulted each other, checked on each other’s welfare and sought help from each other. Participants in both CHCs reported having regular interactions with each other. They interacted through social media (WhatsApp©) and/or meetings that involved reviewing monthly community health service delivery reports submitted by CHPs, planning for community dialogue days, and supervision of CHPs. Members of both CHCs indicated that communication within their teams was good.

Team members in both CHCs regarded their respective CHAs as their “bosses” – rather than fellow CHC members. According to the CHC guidelines, CHAs are supposed to be Secretaries to the CHCs as one female participant responded:

“He is our boss [referring to the CHA]. When we put together the report, he is the one we forward it to. He is our boss”. (Female member, well-functioning CHC, SSI)

Communication to and from the CHA, in most cases, was only through the CHC chairpersons.

Being from the same community, CHC members reported having reciprocal and frequent communication with each other since their relationships were largely personal. Besides the monthly or quarterly scheduled meetings on community health matters, CHC members frequently interacted with each other in social meetings, such as fundraising events, funeral committees, neighborhood security meetings, merry-go-round savings groups, and community-based organization (CBO) meetings. Three participants described their CHC team as “friends” or “family”.

“You know when you live with people, personal issues happen, and work-related issues happen. [If] there was a person who, let us say, needs contribution towards a certain something – let us say a hospital bill, a funeral arrangement, a baby shower or a wedding. You know, because we are a group that you are forced to chip in. [...] We are working together and we have come to know each other and we are friends.” (Male member, well-functioning CHC, SSI)

None of our participants from either CHC reported whether or how they checked each other’s work. Regarding interactions about community health services, CHC members in the well-functioning CHC who also served as CHPs (*n* = 5 of 9) interacted with each other on a monthly basis, compared to quarterly interactions with CHC members that were not CHPs. Monthly interactions between CHC members that were CHPs were mainly about brainstorming on how to resolve conflicts with community members that declined visits by CHPs and addressing poor performance among CHPs.

### Mutual trust

We asked participants about trust and respect within their teams. Participants referred to the terms ‘trust’ and ‘respect’ synonymously. CHC members believed that no CHC member would breach trust by disclosing confidential information that they shared among themselves or by a community member. Most participants indicated that trust and respect were the social “glue” that bound them together as a team:

“Yeah, about that [respect]… even when we began, we said to each other that: ‘No one here has come from their mother’s house, we are all adults.’ I have come from my own home, and so has everyone. We must respect each other. We should all be one.” (Female member, well-functioning CHC, SSI)

### Decision-making

We explored the processes that CHCs used during decision-making, planning, and dividing team tasks. Most participants reported that decision-making and planning within the CHCs was democratic. Six participants from both committees reported that all CHC members got a chance to voice their ideas and opinions, followed by discussions that allowed participation by all team members. Final decisions were usually arrived at through voting. However, when it came to assigning tasks, three participants in the well-functioning CHC mentioned that favoritism created tension among CHC members. During the SSIs, we learned that the chairperson of the well-functioning CHC, who also served as the secretary, selectively assigned community health-related tasks, especially where CHPs were required to implement health activities such as immunization campaigns. CHC members who were not CHPs complained about being sidelined during allocation of community health-related tasks.

“The Chair says we have already chosen three people, they have already gone. Another [job] comes, like polio vaccination. [...] You take the same people again. We have to ask why? Why are you segregating some people?” (Male member, well-functioning CHC, SSI)

### Team cohesion and collectiveness

We asked CHC members about their personal interests, commitment to the team and willingness to work with each other. Participants in both CHCs reported that they had difficulties committing to serve in the CHCs, mainly because this voluntary role had no financial incentives.

“I don’t want – let me just quit, after all I’m not gaining anything, I’m not paid, so most of them dropped out because of that.” (Female member, moderately functioning CHC, SSI)

Participants reported that a lack of financial incentives contributed to a high drop-out of members, especially in the moderately-functioning CHC. We did not establish specific drop-out rates during the SSIs and group discussions. We observed that most CHC members who remained in the moderately functioning CHC were those that doubled up as CHPs.

While exploring how both CHCs maintained team cohesion, we established during the SSIs and group discussions that members of both CHCs had transformed the CHC into merry-go-round savings groups and a community-based organization (CBO). The well-functioning CHC was a formally registered CBO that implemented income-generating initiatives, such as soap making and selling poultry in the community. As merry-go-round savings groups, members of both CHCs collected money regularly (monthly or weekly) to form a pool of money where members would borrow and pay back into the pool.

CHC members in our study related more with the leadership titles in their merry-go-round savings groups or CBO than the leadership titles in the CHC. For example, the title “Assistant Chairman” of the well-functioning CHC was actually a title in the CBO. The Chairpersons of both CHCs in this study were also Chairpersons in their merry-go-round savings group and CBO.

Team cohesion in both CHCs was sustained entirely by the CBO and merry-go-round savings group activities and not by the CHC responsibilities. From our observations, community health oversight roles in both CHCs were secondary to the CBO or merry-go-round savings groups activities, as illustrated in the excerpt below from an SSI:

Moderator: Okay. So as to succeed as a team, what do you think needs to be put in place?

Participant: We usually have a merry-go-round [refers to contribution of money that is pooled] and we meet all the time.

Moderator: Okay. Is that what brings you together?

Participant: It really brings us together because you know people come to the merry go round so as to be able to win the money.

Moderator: Okay, Okay. Is there any other thing that brings you together as a group?

Participant: Just that merry go round. (Female member, moderately functioning CHC, SSI)

Two participants from the moderately functioning CHC revealed that political differences and disagreements around election periods contributed to lack of cohesion among members. For example, differing political views and inclinations sometimes created tension between some team members, which affected how they interacted with each other. During an informal conversation with a member of the moderately functioning CHC, it was said that one member supported a politician who sought to disband the current CHC and impose new team members from one ethnic community. Members, however, denied the existence of tensions due to politics when we explored this topic during group discussions.

### Team motivation

In this theme, we assessed how CHC members encouraged each other to undertake their roles. Members of the moderately functioning CHC stated that the CHA was their primary motivating factor and was the main reason for the CHC’s continued existence:

“The CHA is the one keeping us together. Our CHA tries. When she sees that we are sleeping, she wakes us up quickly. That is why we do not fall flat, so to speak”. (Female member, moderately well-functioning CHC, SSI)

Some CHC team members in the moderately functioning CHC reported that they motivated each other to continue overseeing community health services as a service to God. On the other hand, the merry-go-round savings group and income-generating initiatives in the well-functioning CHC were key motivators for CHC members to stay in the team.

We identified two contextual factors that influenced motivation, especially in the well-functioning CHC. First, CHC members that doubled up as CHPs often received stipends whenever they were called upon to deliver health promotion services in their community. This made the non-CHP members envious and demotivated. Lack of remuneration or stipends to serve as CHC members came up as a source of demotivation for all CHC members as one CHC member stated:

“Salary…Yes we are volunteers, but you can’t be a volunteer for a whole year without money. You would put in more effort if you were to know that there is something that you are getting or… During action days, when you go, you don’t even get something to take some tea [referring to a financial incentive] …” (Female member, moderately-functioning CHC, SSI)

Second, CHCs perceived themselves as “bosses” to CHPs. They were, however, not involved by CHAs whenever CHPs were invited for training or engaged in health promotion activities. This made them feel unrecognized, as expressed by a discussant:

Participant 1: Our prayer is that CHCs be recognized too.

Participant 2: Yeah, because they don’t feature anywhere.

Participant 3: It is like they get lost somewhere in the structure.

Participant 1: They are there, but they get lost somewhere…

Participant 3: They get lost somewhere in the structure.

(Male and female participants, well-functioning CHC, group discussion)

CHC members believed that their performance as a team would be better if they were recognized by CHAs and managers. Once more, CHC members stated that CHAs were their bosses and it was the CHAs who motivated them to continue working as CHC members. None of the CHC members mentioned how CHAs recognized or showed their appreciation to them as a team.

### Team goals and objectives

Individual team members in both CHCs had varying perceptions of team goals. Among most members of the well-functioning CHC, the main team goal seemed to be about establishing a self-sustaining CBO that implemented profitable income-generating activities, while more members in the moderately functioning CHC perceived the team’s objective as supervising CHPs.

Supervision was interpreted by CHC members as monitoring the number of households that CHPs visited and checking the completeness of CHP monthly service delivery reports that were submitted to the CHA. Out of all participants, three participants perceived themselves primarily as CHPs and all the other roles in their team as secondary as illustrated in this quote:

“I remind them: You people you remember you are CHCs, and we need a meeting again with you, you see. They are more CHPs than CHCs.**”** (Male member, well-functioning CHC, SSI)

### Conflict resolution and management

In this theme, we explored processes that CHCs used to resolve and manage conflict. Our exploration of how CHCs resolved conflicts, such as favoritism, revealed that both teams had a sense of camaraderie that made it easy to avoid conflicts between team members. In the event of conflict, team members in both CHCs reported that they first tried to address the issue among themselves without involving “external” parties. Five participants reported that in such cases, they all come together, discuss, and resolve the conflict.

“You know that people who are working together can’t fail to quarrel a bit. [...] We sit down as a CHC, talk it out, so that even when these CHPs look at us, they see that we are united.” (Female member, moderately functioning CHC, SSI)

If CHCs were unable to resolve a conflict, as a team, they involved the CHA – who is officially part of the team but our data show that the CHAs were perceived to be outsiders by CHC members and the CHCs stopped calling the CHAs for their meetings, as one male participant stated during an interview:

“The CHA now… You know, when we were being taught, it was said that the CHA needs to be present in CHC meetings as she [referring to a CHA] was to be one of the CHC members. Whatever we were to discuss, she was to be the secretary and take the minutes. It reached a point where it is like we didn’t have that anymore as the CHA wasn’t being called” (Male member, moderately functioning CHC, SSI)

However, CHC members reported requesting the CHAs to assist them in arbitrating conflicts among team members. CHC members reported that they mainly asked CHAs to help in resolving the allocation of roles in community health interventions, especially those that involved incentives such as allowances and training, as represented in this narrative.

“But something may come up, and they [referring to CHC members who are not CHPs] think that this one may benefit more than this one. But our CHA really tries, when some work comes up, he calls them. We involve them in there [referring to the CHA].” (Female member, well-functioning CHC, SSI)

### Triangulation of research findings

During the group discussion with four CHAs in the study area, participants recommended that for CHCs to work as teams, they required capacity building to understand their roles and to differentiate them from those of CHPs. Secondly, CHAs recommended a review of the CHC training curriculum because it did not sufficiently prepare CHCs for their roles as a team.

## Discussion

In this qualitative case study, we examined teamwork among CHCs and investigated the internal factors that influence teamwork among CHC members in two urban informal settlements using the theoretical framework developed by Yeboah et al. (2013). Our study found that CHC team members perceived their relationships as trusting and respectful, with regular interactions and communication due to their constant proximity as friends and neighbors. Despite CHAs officially being committee members themselves, CHC members looked to them as their supervisors and "bosses," with CHAs perceiving their role as supervisors of CHCs. Our study also revealed that CHC team members focused more on the merry-go-round saving groups or income-generating CBO activities, relegating the CHC's role of overseeing community health services to secondary functions. Complaints of favoritism arose from CHC members who were not CHPs whenever team members who were CHPs received stipends when assigned health promotion tasks in the community.

This study opened the "black box" of CHCs' teamwork [27], which is crucial as the quality of teamwork is linked to the quality and safety of health care programs [28]. In primary care settings, teamwork among community health workers and health professionals decreases disparities in access to health services and improves health-related outcomes [29]. Therefore, understanding teamwork in CHCs is vital for health managers to gain ideas on how to enhance synergy, collective vision, and cohesion among the members who represent different groups in their community. In our observations and analysis, we found no discernible differences in the internal factors that influence teamwork between the well-functioning and moderately functioning CHCs, except for the number of members in each committee. The well-functioning CHCs had more members than the moderately functioning ones. It is possible that health managers labeled the moderately functioning CHCs as such because the smaller number of members may have hindered their ability to carry out their expected roles effectively. Further research could investigate any subtle differences in teamwork between functional and sub-optimally functional CHCs.

Below, we will synthesize the underlying factors that influenced teamwork among CHC members, and will discuss the influence of elites in the community, power dynamics and capacity development on teamwork.

### Dominance of community elite team members in influencing team goals

CHC team members in our study were predominantly community leaders and business owners in the urban informal settlements where they lived. As leaders, they wielded power and influence in their communities and may therefore be perceived as elites [30]. Our findings resonate with those of Carrasco & Bilal (2016), who observed that CBOs and merry-go-round savings groups are predominantly composed of local elites [31]. It is possible that the CHC teams that doubled up as CBOs or merry-go-round saving groups in our study sites reflected the interests of the elite members [30, 32, 33]. These elites might have played an important role in transforming the identity and “shared vision” of the CHCs into those of merry-go-round savings groups or CBOs. We argue that elites may have been motivated to subvert the objectives of the CHC teams into merry-go-round saving groups or CBOs to suit their individual interests at the expense of the community health objectives [27, 32, 34, 35]. Merry-go-round saving groups are popular in resource-poor settings for assisting group members to save regularly and provide a platform that allows them to borrow money to meet essential day-to-day needs and to meet unexpected expenses [31, 36]. Merry-go-round savings groups also provide an informal source of credit for households and small enterprises [36]. Literature shows that it is common for community-level groups that are formed to perform community-level health functions to evolve into CBOs [37]. In our study, CHCs evolved into an income-generating CBO and merry-go-round savings group.

Transforming CHC teams into merry-go-round saving groups, where members contribute money regularly, might have transformed CHCs into “invited spaces” that are created by elites [38]. Contrary to community health strategy intentions, the two CHCs in our study cannot be regarded as social spaces where also marginalized community members, with little or no disposable income, could practice equitable participation in the oversight of community health services [39].

### Power dynamics and teamwork

Power dynamics among team members and with the CHAs were evident in our study. CHCs are social spaces where power relations among team members and with other health system actors play out [39]. Like other health care teams, CHCs are congenial “natural communities”, where power imbalances also play out [27]. These power imbalances influence the horizontal interactions between members.

CHC team members who served as CHPs and were also community leaders and business persons wielded their power by inviting fellow CHPs in the CHC teams to participate in health promotion activities and leaving out team members who were not CHPs. Exercise of this power to select who participates in activities that had financial incentives attached to them created conflict in the teams. It is evident that these power imbalances have the potential to cripple participation in CHCs, if not well managed. CHAs who are government representatives exercised “institutional” power over CHC members. This may explain why CHC members referred to them as their “bosses”. Exercise of institutional power over CHC teams by government representatives (who are on paper but not in practice part of these teams) may have diminished the autonomy of CHCs in making decisions about their priorities. Here we note that the “institutional power” wielded by CHAs could override the power wielded by the influential community elite.

### Capacity development in teamwork

Our study unraveled how inadequate capacity development of CHCs on their functions and teamwork contributed to variations in team goals and deviation from their core mandate, which is to oversee community health services. Teamwork for CHCs is an integral competency that requires to be continuously strengthened for these teams to effectively oversee community health services [10]. Capacity development has been used both as an individual- and team-level intervention to improve teamwork at individual level (skills and attitudes), team level (efficiency), and organizational level (safety culture) [40]. Our findings indicate that CHC members had different goals from what is described in the community health strategy. Lack of a clear understanding of roles and functions in a team often lead to friction among members, confusion, wasted resources, and effort [21, 41]. Lack of understanding of the CHC goal among team members may be attributed to inadequate training. Ongoing training needs to address specific skill sets required for the teams to work effectively and “soft skills” such as communication and conflict resolution strategies [42].

Strengthening of teamwork in health systems has been implemented widely and has been successful in improving individual (attitudes) and team-level (efficiency) competencies. Capacity strengthening can be done continuously through on-the-job training, supervision and regular team briefings which should be integrated into managers’ work plans and be evaluated. Capacity strengthening requires strong leadership from CHAs and health managers who are responsible for community health services at national and sub-national levels of Kenya’s health system [28, 40, 43].

One limitation of our case study is that we collected data from two urban informal settlements, which is therefore not representative of CHCs in other regions. However, our study provides a stepping stone for further exploration of how to improve teamwork in CHCs. Our study design did not enable us to explore in depth the political and intersecting factors (gender, age, socio-economic, ethnic) that influence teamwork, because CHC members were not willing to discuss these issues with us. The CHCs in our study were predominantly women. This may have skewed the perspectives. However, recent research demonstrated that CHPs in urban informal settlements are predominantly composed of women [44]. We recommend applying ethnographic methods to explore these issues. We furthermore emphasize the importance of assessing software attributes of teams using robust frameworks that assess interactions between individuals in teams.

## Conclusion

This study sheds light on the intricate social processes that impact teamwork within CHCs. Despite the small-scale nature of our study, it reveals the dynamics and social nature of CHCs. Our findings suggest that when leadership and direction are absent in committing to CHC goals, team building, capacity strengthening, and incentives for CHCs, they may adapt or transform into groups that serve other collective interests and needs of the members, such as income-generating savings groups. As a result, the teamwork that would have supported community health services is redirected to other common interests of the team. To harness the benefits of teamwork in community health services, it is essential for health managers and CHAs to receive training on addressing the influence of community elites on CHCs, building effective teams, and fostering teamwork. Additionally, we suggest including a module on developing teamwork in the manual for training CHCs, as such a module is currently not available. This training content should form the basis for continuous support and appraisal of CHCs' teamwork as part of routine performance reviews.

## Data Availability

The data presented in this study are available on reasonable request from the corresponding author. The data are not publicly available due to the need to protect the confidentiality of participants involved in the study.

## Abbreviations

CHA: Community Health Assistants
CHC: Community Health Committees
CHP: Community Health Promoters
CBO: Community-Based Organization
FGD: Focus Group Discussion
MoH: Ministry of Health
NGO: Non-government Organization
SSI: Semi structured interview
WHO: World Health Organization
